# The Influence of Laser Shock Peening on the Microstructure and Mechanical Properties of AH32 Steel

**DOI:** 10.3390/ma18204679

**Published:** 2025-10-12

**Authors:** Xu Pei, Yiming Shen, Zhaomei Xu, Pengfei Li, Yuchun Peng

**Affiliations:** 1 Faculty of Mechanical and Material Engineering, Huaiyin Institute of Technology, Huai’an 223003, China; xuzhaomei@hyit.edu.cn; 2 School of Mechanical Engineering, Jiangsu University, Zhenjiang 212013, China; 2212303040@stmail.ujs.edu.cn; 3 Department of Smart Agriculture and Engineering, Wenzhou Vocational College of Science and Technology, Wenzhou 325006, China; 2111903008@stmail.ujs.edu.cn

**Keywords:** AH32, laser shock peening, tensile strength, tensile strength

## Abstract

The mechanical integrity of shipbuilding steel under demanding maritime service conditions is a pivotal factor for ensuring the structural safety and operational longevity of vessels. This research employs laser shock peening (LSP) to augment the surface performance of AH32 steel and carries out a comprehensive analysis of the influence and underlying mechanisms of LSP on both the microstructural evolution and mechanical properties of the material. The results indicate that the LSP treatment successfully introduced a high magnitude residual compressive stress (−162 MPa) at the surface of AH32 steel. Additionally, the surface hardness of LSP-1 and LSP-2 increased by 7.3% and 14.7%, respectively. The tensile test results indicate that Sample LSP-2 achieved a 25.8% improvement in elongation while exhibiting only a 5.9% reduction in ultimate tensile strength. Friction and wear tests demonstrated that the average coefficient of friction for the samples treated with LSP decreased by approximately 18%, while the wear rate reduced significantly by over 40%.

## 1. Introduction

The service environment of marine engineering equipment and deep-sea vessels is extremely harsh, with structural materials subjected to a combination of factors over an extended period, including alternating loads, seawater corrosion, and frictional wear caused by sediment and ice [1]. Surface wear is among the primary causes of structural performance degradation, reduced lifespan, and even catastrophic accidents in the aforementioned contexts [2]. Therefore, effectively enhancing the mechanical properties and wear resistance of key metal structural components has become a core issue in ensuring the safety and reliability of marine equipment [3].

AH32 steel, as a classic high-strength low-alloy steel (HSLA) for hulls, is widely used in critical areas such as ship shells, decks, and offshore platforms due to its excellent overall mechanical properties, weldability, and seawater corrosion resistance [4]. However, under long-term harsh marine conditions, the surface of AH32 steel components remains a weak link for the initiation of fatigue cracks and wear. Traditional surface modification techniques, such as heat treatment and shot peening [5], while capable of improving surface properties to some extent, often have limitations such as shallow modified layers, large heat-affected zones, and susceptibility to deformation or deterioration of surface roughness. Therefore, developing an efficient, controllable, and non-thermal surface strengthening technology is of significant practical engineering importance for further enhancing the service performance of AH32 steel [6].

LSP is recognized as a technique capable of imposing severe plastic deformation on material surfaces at ultra-high strain rates, thereby imparting significant compressive residual stresses and substantial plastic strain [7]. Bu et al. [8] investigated the effect of laser shock peening (LSP) on the microstructural evolution and mechanical properties of IN718. The results indicated that LSP induced plastic deformation and grain refinement in the near-surface region of the specimens. Samples treated with LSP at a laser pulse energy of 9 J exhibited a 9.3% increase in tensile strength and a 9.0% improvement in elongation. In a related study, Wu et al. [9] employed LSP to fabricate a gradient nanostructure along the depth direction in the AM50 magnesium alloy. Their findings demonstrated an approximately 12% enhancement in ultimate tensile strength, albeit with a corresponding reduction in tensile ductility of about 3%. Ning et al. [10] studied the wear performance of the IN738 high-temperature alloy manufactured by electron beam powder bed fusion (EBPBF) under 600 °C after LSP. The results indicated that, compared to the original samples, the average coefficient of friction (COF) and average wear rate of the composite materials were reduced by 23.6% and 73.6%, respectively. Li et al. [11] investigated the impact of multiple LSP treatments on the wear resistance of 17-4 PH stainless steel. The results indicated that as the number of peening cycles increased, the surface grain refinement became evident, leading to an improvement in the wear resistance of the material.

Based on the above analysis, this study aims to systematically and comprehensively investigate the effects and mechanisms of LSP treatment on the microstructure and tribological performance of AH32 marine steel. Residual stresses, surface texture, and microhardness were assessed, while the material’s microstructure was analyzed through scanning electron microscopy (SEM). Additionally, the wear mechanisms were explored through the evolution of the microstructure and the morphology of the worn surface.

## 2. Experimental

### 2.1. Materials and LSP Experiment

The material used in this study is AH32. The elemental composition is shown in Table 1. The steel was produced in an electric arc furnace using scrap steel as the raw material, with additions of Nb and V for grain refinement. After casting, the slabs were rolled at 1100 °C to the target thickness to eliminate casting defects. Following thermomechanical controlled processing (TMCP), no additional heat treatment was applied.

For the LSP process, the laser beam was configured with the following parameters: a wavelength of 1064 nm, pulse width of 8 ns, pulse energy of 1 J, spot diameter of 2 mm, repetitive rate of 2 Hz, and 50% overlap between consecutive spots. Black tape was employed as the absorbing layer for laser energy, and the water layer was used as the transparent confining medium to enhance the plasma pressure generated during LSP. The resulting specimens are referred to as follows: the as-received condition as AR, while those treated with one and two LSP passes are designated as LSP-1 and -2, respectively. The specimen dimensions are 15 mm × 15 mm.

### 2.2. Measurements

The surface roughness evolution of the specimens was measured using a three-dimensional profilometer (VK-X200, Osaka, Japan). The specimen dimensions are 15 mm × 15 mm. The analyzed regions for roughness measurement were 150 × 100 μm^2^. Additionally, this study evaluated the line and surface roughness. The profile roughness was characterized by the arithmetic mean deviation (Ra), while the areal roughness parameters included the arithmetic mean height (Sa), root mean square height (Sq), and maximum height (Sz).

The microstructure of the corroded samples was examined by scanning electron microscopy (SEM, JSM-7800F, Akishima, Japan) equipped with energy-dispersive X-ray spectroscopy (EDS). The specimen dimensions are 10 × 10 mm. Further, 4% nitric acid-ethanol was utilized for etching the samples for 15 s. Electron backscatter diffraction (EBSD) analysis was performed using a JEOL-6500F field emission scanning electron microscope (JSM7800F, Akishima, Japan), the specimen dimensions are 10 × 10 mm. Residual stress measurements were performed using the sin^2^ψ method with an X-350A X-ray stress testing system (ST, Handan, China), which yielded a stress constant of −218 MPa/degree. The 2θ scan commenced at an angle of 161° and concluded at 150°. The phase composition of the samples was determined using a Cu Kα radiation X-ray diffraction (XRD, Bruker D8 Advance, Karlsruhe, Germany) system, with a scanning angle range of 30° to 90° and a scanning speed of 5°/min, employing a step size of 0.02°. The specimen dimensions are 15 mm × 15 mm.

### 2.3. Mechanical Testing

In accordance with the <GB/T 21838.1-2019> standard, Vickers microhardness measurements were conducted on metallographically prepared specimens using a load of 0.5 kg and a dwell time of 15 s. Microhardness measurements were conducted on a micro-Vickers hardness tester (Model 000 ZB, Koliti, Guangzhou, China) with an interval of 10 mm between adjacent indentations to avoid mutual interference. The specimen dimensions are 15 mm × 15 mm.

Tensile tests were performed on an INSTRON 5569 electronic universal testing machine, following the <GB/T 228-2002> standard entitled a gauge length of 11 mm was used, and the tests were carried out at a constant crosshead speed of 1 mm/min. To ensure statistical reliability, three repeat tests were conducted for each material condition. Fractographic analysis of the tensile specimens was subsequently performed using SEM (JSM7800F, Akishima, Tokyo, Japan).

### 2.4. Friction

Friction and wear behavior were evaluated using a Retc universal tribometer (Retc, San Jose, CA, USA), with a 5 mm diameter Si_3_N_4_ ball employed as the counterface material. Tests were carried out under a normal load of 10 N and a rotational speed of 500 revolutions per minute with a wear track radius of 3 mm for a total duration of 30 min. The specimen dimensions are 15 mm × 15 mm. The mass of the samples before and after the friction wear tests was determined using a BSM 2204 analytical balance. To ensure accuracy, three repeated measurements were taken both pre- and post-wear, and the average values were reported.

## 3. Result

### 3.1. Roughness

**Figure 1:**
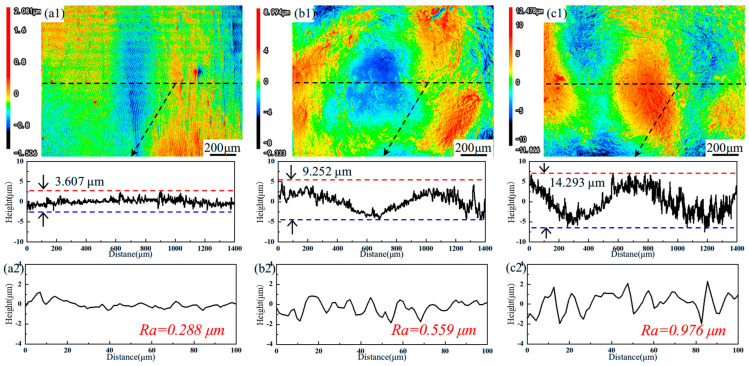
Surface images and line roughness profiles of (**a**) AR, (**b**) LSP-1, and (**c**) LSP-2 samples.

Figure 1 presents the surface profiles of the samples. As shown in Figure 1(a1), the AR sample exhibits a relatively even topography, while the LSP-treated sample shows discernible surface undulations, a characteristic morphological outcome of the LSP process, as evident in Figure 1(b1,c1). The profile height of the LSP-1 sample is approximately 9.252 μm, while the LSP-2 sample, derived from the LSP-1 sample, has a profile height of about 14.293 μm. To quantitatively evaluate the surface roughness of all samples, the roughness parameter Ra is presented in Figure 1(a2–c2). A smaller Ra value indicates a smoother surface, and conversely, a larger Ra value suggests increased roughness. The increase in surface roughness from sample LSP-1 to LSP-2 indicates that repeated laser shock peening leads to a cumulative increase in surface roughness [12].

**Figure 2:**
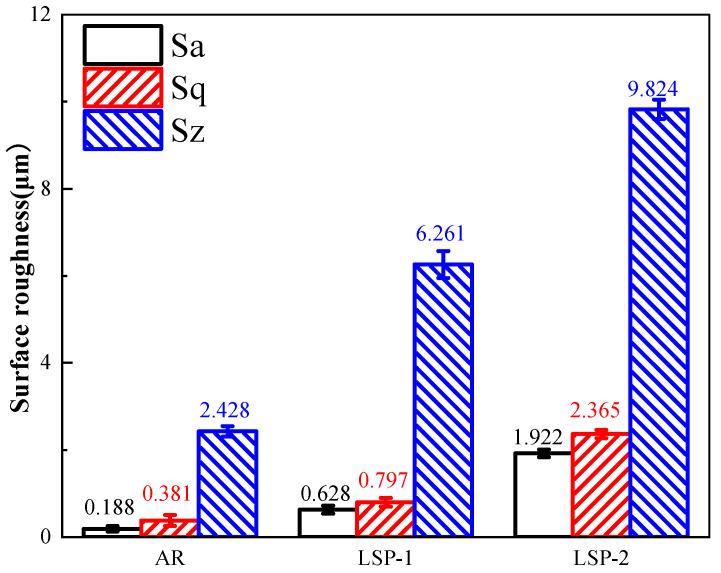
Surface roughness parameters measured on the three samples.

Surface roughness parameters (Sa, Sq, and Sz) were measured to quantitatively evaluate all specimens, with the results presented in Figure 2. All LSP-treated samples exhibited significant surface roughening. The roughness parameters showed a consistent increasing trend from LSP-1 to LSP-2. Specifically, the Sz value increased from 2.428 μm for the AR sample to 6.261 μm for LSP-1 and further to 9.824 μm for LSP-2.

### 3.2. XRD and Residual Stress Analysis

**Figure 3:**
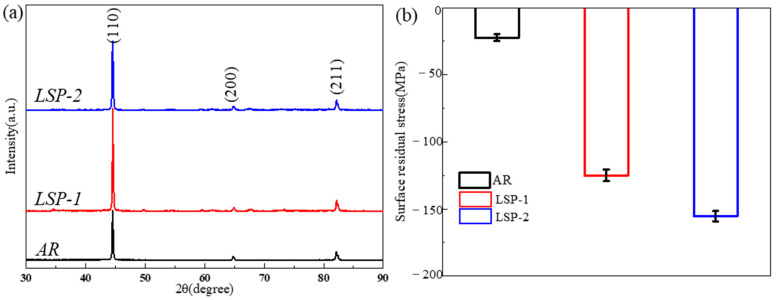
(**a**) X-ray diffraction patterns; (**b**) corresponding surface residual stress measurements for each sample condition.

The XRD patterns of the samples are presented in Figure 3a. In accordance with Bragg’s law [13], this causes a corresponding rightward shift in the XRD diffraction peaks. The full width at half maximum (FWHM) values of the diffraction peaks for all samples are summarized in Table 2. The FWHM of the LSP-1 sample remains largely unchanged, whereas that of the LSP-2 sample demonstrates a pronounced increase, suggesting considerable grain refinement induced by the LSP process.

Figure 3b presents the residual stress measurements on the surface of the AR sample and the two LSP-treated samples. The results indicate that the AR sample exhibits an average residual compressive stress of −17 MPa. In contrast, the LSP-processed samples, subjected to high-energy laser pulses, show a significant increase in compressive residual stress (CRS), with average values reaching −133 MPa for LSP-1 and −162 MPa for LSP-2.

### 3.3. Microstructural Analysis

**Figure 4:**
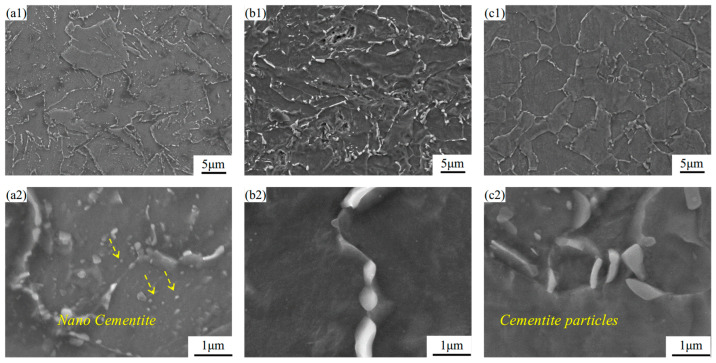
SEM morphology of the AR sample and LSP samples: (**a1**,**a2**) AR, (**b1**,**b2**) LSP-1, and (**c1**,**c2**) LSP-2.

Figure 4b,c displays the deformed microstructure after LSP treatment, which consists of ferrite and granular cementite. Following LSP, a significant reduction in cementite particles within grain boundaries is observed, as shown in Figure 4(b2). Furthermore, under the influence of laser shock, continuous precipitation of cementite particles along grain boundaries is evident in Figure 4(b1), which is consistent with the findings reported by Karimi [14]. Meanwhile, the size of cementite in the LSP-1 sample increases to approximately 0.67–1.08 μm. In the LSP-2 sample, the cementite particles measure about 0.79–0.88 μm.

**Figure 5:**
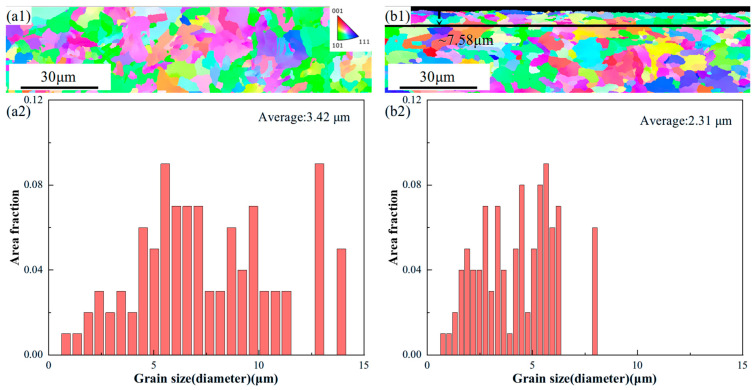
EBSD morphologies of samples: (**a1**) AR, (**a2**) the corresponding grain size distributions with a1, (**b1**) LSP-2, (**b2**) the corresponding grain size distributions with b1.

Figure 5 presents the EBSD morphologies and corresponding grain size statistics for the AR and LSP-2 samples. Both samples exhibit random crystallographic orientations without preferred texture components. As shown in Figure 5(a2), the AR sample has an average grain size of 3.42 μm. After LSP-2 treatment, the affected depth reaches approximately 7.58 μm, within which the average grain size is refined to approximately 2.31 μm, as depicted in Figure 5(b1). Compared with the AR sample, this represents a grain refinement of 32.4%.

### 3.4. Microhardness and Tensile Properties

**Figure 6:**
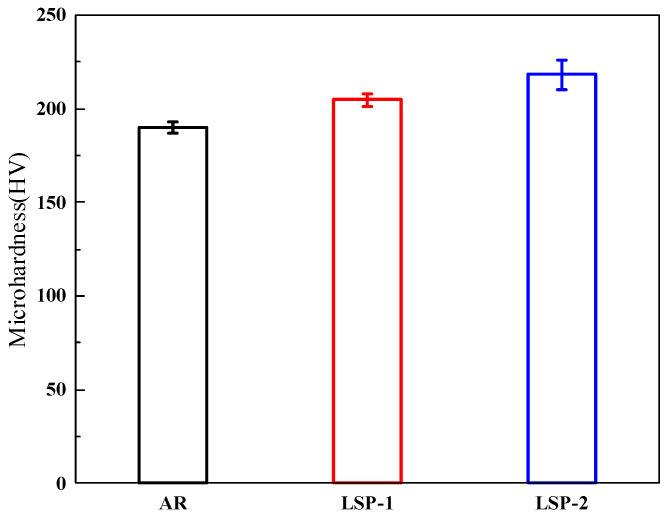
Microhardness of three samples.

Figure 6 compares the microhardness measured by the Vickers method for the AR and LSPed samples. To enhance testing accuracy, each sample was tested five times. The hardness of the AR sample is 190.2 HV, while the hardness values for the LSP-1 and LSP-2 samples are 204.7 HV and 218.3 HV, respectively, representing increases of 7.3% and 14.7%, respectively. According to previous studies [15], LSP samples undergo significant plastic deformation, which induces work hardening on the surface of the steel, resulting in an increase in hardness.

**Figure 7:**
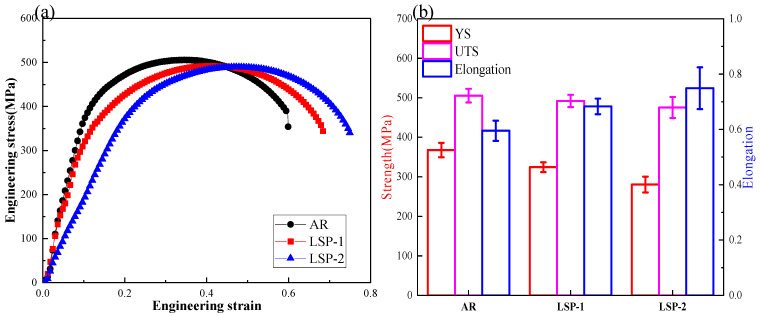
Mechanical properties of the three samples. (**a**) Engineering stress−strain curves. (**b**) Ultimate tensile strength, yield strength, and elongation.

Figure 7 displays the ultimate tensile strength (UTS), yield strength (YS), and elongation after fracture for the AR and LSPed samples. The mechanical properties and error bars in Figure 7b are based on the average values and standard deviations obtained from three measurements. The AH32 steel exhibits a significant enhancement in ductility following LSP treatment, with elongation increasing from 59.5% in the AR condition to 68.3% after LSP-1, and further to 74.9% after LSP-2. In contrast, the yield strength decreased from 367.8 MPa (AR) to 324.4 MPa (LSP-1) and 280.5 MPa (LSP-2). Notably, the ultimate tensile strength (UTS) remained largely unaffected, decreasing only marginally from 505.3 MPa (AR) to 475.3 MPa (LSP-2), as illustrated in Figure 7b.

**Figure 8:**
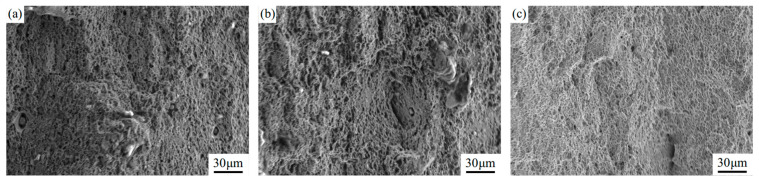
Tensile fracture morphologies of different samples: (**a**) AR, (**b**) LSP-1, and (**c**) LSP-2.

The fracture morphology after tensile testing is shown in Figure 8. The surface of the AH32 sample is covered with dimples of various sizes, along with some inclusions. In contrast, the LSP-2 sample exhibits a greater number of finer dimples, demonstrating typical ductile fracture behavior. LSP treatment leads to grain refinement in the surface layer, resulting in a strengthened hardened layer. The presence of smaller dimples and the variation in morphology on the fracture surface confirm the increase in material strength, as the formation of dimples requires greater stress [16].

### 3.5. Friction

**Figure 9:**
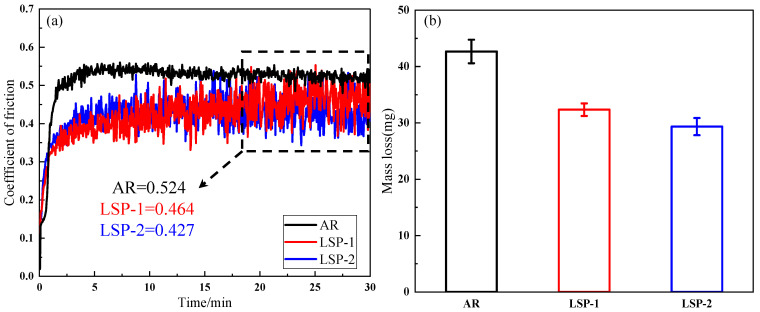
(**a**) The variation curve of the friction coefficient. (**b**) The average mass loss.

Figure 9 depicts the evolution of the coefficient of friction (COF) with sliding time for the three sample conditions. The COF curve in Figure 9a can be divided into three stages, characterized by the trend of COF changes. The first stage represents the running-in phase, dominated by the peaks of roughness at the contact interface between the sample surface and the paired ball, leading to a rapid increase and significant fluctuations in the friction coefficient [17]. The second stage corresponds to a period of severe wear, during which intense wear occurs on the sample surface [18]. Over time, a stable contact forms between the paired ball and the sample surface, resulting in a downward trend in the friction coefficient. The third stage is the stable friction phase, where the COF reaches a constant value, with the AR sample having a coefficient of 0.524, while LSP-1 and LSP-2 exhibit values of 0.464 and 0.427, respectively. Figure 9b shows the mass loss after friction and wear for the three samples, with the AR sample exhibiting the highest mass loss of 42.6 mg, while LSP-2 shows the lowest mass loss of 29.3 mg.

**Figure 10:**
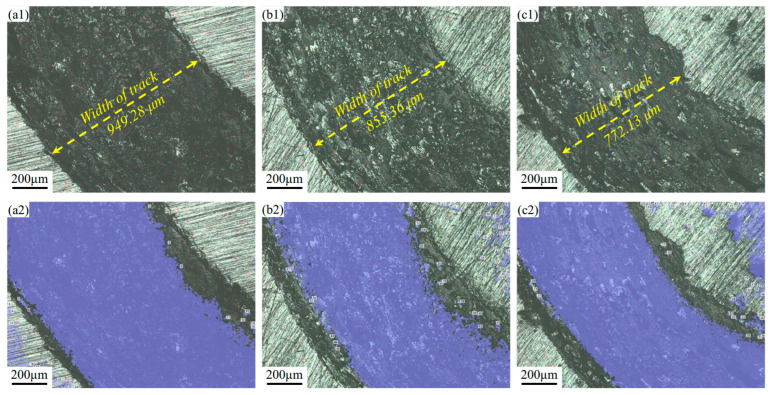
Two-dimensional morphology characterization of the wear tracks and the corresponding quantitative wear assessments after 30 min of sliding: (**a1**) AR, (**a2**) wear assessments of AR, (**b1**) LSP-1, (**b2**) wear assessments of LSP-1, and (**c1**) LSP-2, and (**c2**) wear assessments of LSP-2.

The wear morphology of different samples is shown in Figure 10, with the total wear volume and area for the selected regions presented in Table 3. The wear track of the AR sample is wider, resulting in a larger wear volume (10,872,236.220 μm^3^) and an area ratio of 71.335%. Additionally, the friction width is the largest at 949.28 μm, as depicted in Figure 10(a1). The wear of the LSP-1 sample is more uniform, with a gradual transition in wear depth. The boundary between the wear area and the substrate is very clear, and the worn surface exhibits slight shallow plowing scratches and minimal debris residue, as shown in Figure 10(b1). The post-wear surface morphology indicates a volume reduction to 9,680,137.442 μm^3^ and a decrease in area ratio to 62.468%. In contrast, the LSP-2 sample exhibits the lowest wear width (772.13 μm) and wear volume at 57.369%. In summary, LSP treatment is beneficial for the wear behavior of AH32.

**Figure 11:**
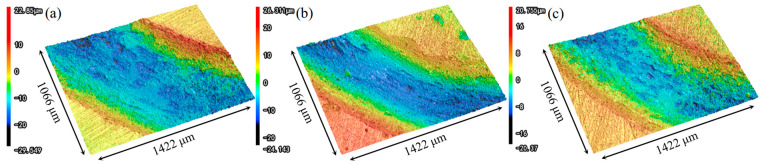
3D profiles of three samples: (**a**) AR, (**b**) LSP-1, and (**c**) LSP-2.

Figure 11 presents the wear morphology of the three sample conditions, wherein Figure 11a–c depict the corresponding wear track profiles. A marked reduction in both the width and depth of the wear tracks is observed in the LSP-treated samples compared to AR.

**Figure 12:**
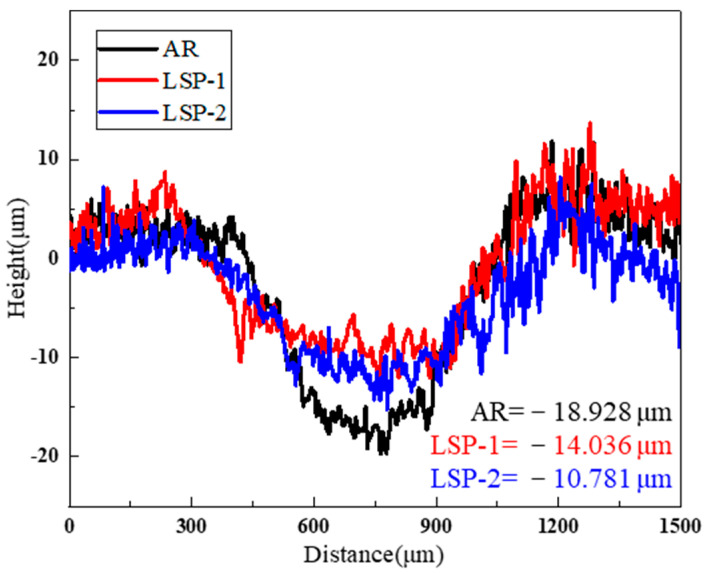
Comparison of the worn cross-section morphology of three samples.

Figure 12 exhibits the cross-sectional profiles of the wear tracks, and the findings align consistently with the topographic features observed in the three-dimensional morphological analysis. Under the same testing time and load, it is straightforward to analyze the maximum depth of the wear zones for the different samples. The AR sample exhibits the maximum wear depth, approximately 18.9 μm. The maximum wear depth for LSP-1 is around 14.1 μm, while LSP-2 shows a further reduction in maximum wear depth to 10.8 μm.

## 4. Discussion

Cementite, serving as a strengthening phase in steel, exhibits nearly negligible toughness, which results in a significant mechanical property mismatch with the surrounding ferrite matrix during plastic deformation. Under external loading, this mismatch leads to pronounced stress concentration at the two-phase interfaces, thereby promoting the initiation of microcracks. Following LSP treatment, the morphology and distribution of cementite are notably optimized—transitioning from a dispersion of fine point-like particles in the AR sample to distinct granular forms in the LSP-2 condition (Figure 4). These granular cementite particles effectively redistribute the high stress originally concentrated at the grain boundaries across numerous fine particles, significantly mitigating the stress concentration intensity [19].

Concurrently, EBSD analysis confirms that LSP induces substantial grain refinement. Specifically, the LSP-2 sample exhibits a 32.5% refinement in surface grain size compared to the AR condition. This microstructural improvement contributed to a 25.8% increase in elongation, while the ultimate tensile strength experienced only a minor reduction of 5.9%. The pronounced grain refinement induced by LSP is identified as one of the primary contributors to the enhanced strength. Consequently, the LSP-treated sample, strengthened by the surface refined grain layer, demonstrates effective simultaneous improvement in both yield strength and ductility [20].

According to previous studies [21], the sliding contact of friction pairs against a material surface generates tensile stress, which can initiate crack formation and lead to surface spalling during repeated friction cycles. However, the CRS field introduced by LSP has been shown to effectively counteract this detrimental tendency [22]. In comparison to the AR sample, both LSP-treated specimens exhibit significantly higher levels of CRS. This enhanced compressive stress state correlates with a reduction in wear rate, mitigated surface spalling (as evidenced in Figure 10), and a consequent further decrease in wear loss. Owing to the repeated laser impacts, the LSP-2 sample possesses a more pronounced CRS, as shown in Figure 3b, which contributes to its improved tribological performance and lower COF.

**Figure 13:**
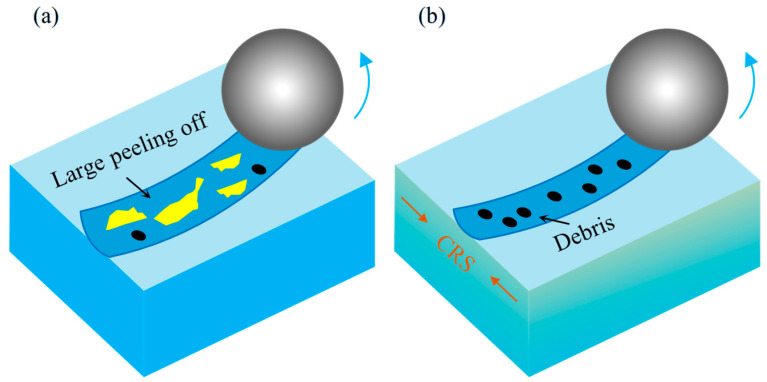
Schematic diagram of the wear mechanism: (**a**) AR and (**b**) LSP-2.

Building upon the foregoing analysis, Figure 13 schematically illustrates the wear mechanisms operative in the AR and LSP-treated samples. As illustrated in Figure 13a, the absence of a pre-existing work-hardened layer in the AR sample, combined with its minimal hardness, renders it susceptible to substantial plastic deformation under wear-induced stress. The predominant wear mechanism identified is adhesive wear, a conclusion further supported by the distinct spalling and delamination features visible in Figure 10(a1). The deformation layer induced by wear is relatively shallow, and the degree of work hardening remains minimal. This microstructural state renders the surface material prone to adhesive material transfer and spalling caused by the counter friction pair, as conceptually summarized in Figure 13a.

As indicated in Figure 6, the surface hardness of AH32 steel is significantly enhanced following LSP treatment. This improved surface hardness effectively inhibits the formation of wear debris during sliding contact. The hardened surface reduces the tendency of debris to embed into the material, while any spalled particles are further fragmented into finer debris under high contact temperatures, as illustrated in Figure 13b. Throughout the wear process, such debris acts as third-body abrasives, simultaneously impinging upon both the counterface and the sample surface. Abrasive wear emerges as the predominant mechanism in the LSP-2 specimen. This shift in wear behavior contributes to the decline in the average COF, as clearly indicated in Figure 9(a1).

## 5. Conclusions

(1) The plastic deformation induced by LSP increases the surface roughness of AH32, while also inducing high-magnitude residual stress on the surface. The residual stress for the LSP-2 sample reaches −162 MPa.
(2) The typical microstructure of AH32 steel mainly consists of ferrite and carbides. The microstructural evolution after LSP treatment is primarily characterized by a significant reduction in cementite within the grain boundaries, while cementite particles precipitate along the boundaries of the grains. The LSP-2 sample exhibited an affected layer depth of approximately 7.58 μm, which was accompanied by a 32.4% refinement in grain size compared to the AR sample.
(3) After LSP treatment, the surface hardness of the samples increased by 7.3% and 14.7%, respectively. The tensile results indicate that LSP-2 exhibits a 25.8% enhancement in elongation while demonstrating only a marginal 5.9% reduction in ultimate tensile strength.
(4) Compared to the AR samples, the LSP samples exhibited lower friction coefficients and wear rates, with the wear coefficients decreasing by 11.4% and 18.5%, respectively.

## Figures and Tables

**Table 1:** The elemental composition of AH32.

	C	S	P	Cr	Ni	Mn	Fe
AH32	≤0.18	≤0.5	≤0.03	≤0.2	≤0.4	0.9–1.6	Balanced

**Table 2:** Comparison of FWHM.

Lattice Plane	AR	LSP-1	LSP-2
(110)	0.208	0.223	0.241
(200)	0.342	0.346	0.416
(211)	0.433	0.442	0.473

**Table 3:** Comparison of worn volume, cross-section area, and area ration.

Samples	Total Worn Volume/μm^3^	Cross-Section Area/μm^2^	Area Ration/%
AR	10,872,236.220	1,085,310.616	71.335
LSP-1	9,680,137.442	920,414.825	62.468
LSP-2	5,680,900.656	872,829.626	57.369

## Data Availability

The raw data supporting the conclusions of this article will be made available by the authors on request.
